# Infantile Mortality and Amateur Hygienists

**Published:** 1911-02-18

**Authors:** 


### INFANTILE MORTALITY AND AMATEUR HYGIENISTS.

MEASURES having for their aim the reduction of infantile mortality seem likely to become as popular with amateur sanitarians as those which are directed to the prevention of tuberculosis. Well directed and subordinated to expert professional control, this public enthusiasm is one of the greatest forces of sanitary reform. It is needed to overcome the obstructive inertia of the established order, and should never be wasted in ineffectual and useless demonstration of its own activity. Conferences and congresses held for the purpose of improving and diffusing knowledge on questions of State medical interest are undoubtedly valuable. They enable local authorities to become cognisant of the practice and administrative procedure of kindred bodies throughout the country, and of kindred bodies throughout the country, and so to co-ordinate local government that public health administration, while preserving local character and initiative, shall retain such general unity of purpose and practice as is consonant with national government. Conferences are helpful in educating the lay members of public health committees to the need for measures only too apt to be treated as the "fads" of the medical profession. They afford an occasion for recording the scientific results of orderly observation and an opportunity for formulating those principles sanctioned by experience which are to be the basis of future action and further modification of practice. For these reasons we approve the holding of conferences and are anxious that nothing shall be done calculated to bring them into disrepute or destroy the good influences which they exercise. The Infantile Mortality Conference of 1906 justified itself, because it focussed opinion and unified practice in a field of preventive medicine in respect of which the national conscience had shown signs of quickening, and diffused a knowledge of the administrative practice initiated by the medical officers of health of the more active local authorities.

We confess, however, to deep disappointment with later conferences, some of which were uncalled for, and even contra-indicated, and which could only have been justified by some brilliant and unexpected turn given to the consolidating phase of administrative action which has now been reached. Some of these conferences have proved to be merely a deluge of talk, monotonously reiterating the obvious and commonplace. Medical officers of health conspicuous by their silence were edified not less by the shrill harangues of their lady sanitary inspectors and health visitors than by the pastoral homilies of municipal Solons. The scene presented was like that of an army in which the generals were being instructed in the art of war by the soldiery and a sprinkling of uninstructed civilians, well intentioned but hopeless. The spectacle of the amateur directing, or attempting to direct, measures aiming at the solution of what are strictly medical problems is one with which we have become only too familiar in this country. We are not prepared to shudder at the phantasm of a medical dictatorship which, with strange obliquity of vision, has been raised in some quarters; but we do think there is a serious menace in the loud braying of unqualified opinion which masquerades as authoritative, and is accepted as such by an uncritical public. The social aspect of the problem of infantile mortality, necessitating as it does the enlistment of a large army of lay workers, official and voluntary, must not be permitted to confuse the essentially medical character of the question. If the efforts to reduce the unnecessarily high mortality of infants are not subjected to medical direction, and the measures adopted are not informed by scientific knowledge, the popular crusade against this serious social blot will become a riot of ignorant fanaticism and culminate in mischievous disappointment.

